# Using Pharmacogenetics of Direct Oral Anticoagulants to Predict Changes in Their Pharmacokinetics and the Risk of Adverse Drug Reactions

**DOI:** 10.3390/biomedicines9050451

**Published:** 2021-04-22

**Authors:** Natalia A. Shnayder, Marina M. Petrova, Pavel A. Shesternya, Alina V. Savinova, Elena N. Bochanova, Olga V. Zimnitskaya, Elena A. Pozhilenkova, Regina F. Nasyrova

**Affiliations:** 1 The Centre of Personalized Psychiatry and Neurology, V. M. Bekhterev National Medical Research Center for Psychiatry and Neurology (V.M. Bekhterev NMRC PN) 3, Bekhterev Str., 192019 Saint-Petersburg, Russia; alina.v.savi@gmail.com; 2 The CoreFacilities Molecular and Cell Technologies, V. F. Voino-Yasenetsky Krasnoyarsk State Medical University (V.F. Voino-YasenetskyKrasSMU) 1, PartizanZheleznyak Str., 660022 Krasnoyarsk, Russia; stk99@yandex.ru (M.M.P.); shesternya75@mail.ru (P.A.S.); bochanova@list.ru (E.N.B.); zvezda_5786@mail.ru (O.V.Z.); elena.a.pozhilenkova@gmail.com (E.A.P.)

**Keywords:** pharmacokinetics, pharmacogenetics, dabigatran, rivaroxaban, apixaban, edoxaban

## Abstract

Dabigatran, rivaroxaban, apixaban, and edoxaban are direct oral anticoagulants (DOACs) that are increasingly used worldwide. Taking into account their widespread use for the prevention of thromboembolism in cardiology, neurology, orthopedics, and coronavirus disease 2019 (COVID 19) as well as their different pharmacokinetics and pharmacogenetics dependence, it is critical to explore new opportunities for DOACs administration and predict their dosage when used as monotherapy or in combination with other drugs. In this review, we describe the details of the relative pharmacogenetics on the pharmacokinetics of DOACs as well as new data concerning the clinical characteristics that predetermine the needed dosage and the risk of adverse drug reactions (ADRs). The usefulness of genetic information before and shortly after the initiation of DOACs is also discussed. The reasons for particular attention to these issues are not only new genetic knowledge and genotyping possibilities, but also the risk of serious ADRs (primarily, gastrointestinal bleeding). Taking into account the effect of the carriership of single nucleotide variants (SNVs) of genes encoding biotransformation enzymes and DOACs metabolism, the use of these measures is important to predict changes in pharmacokinetics and the risk of ADRs in patients with a high risk of thromboembolism who receive anticoagulant therapy.

## 1. Introduction

Thromboembolism (such as stroke and systemic embolism) is a serious complication of non-valvular atrial fibrillation (AF) [1]. Pulmonary embolism (PE) can cause death within the first 14 days after a stroke in 25–50% of cases [2]. In the absence of preventive measures, before widespread use of anticoagulant therapy in clinical practice, the percentage of venous thromboembolic complications in lower limb arthroplasty (deep vein thrombosis and PE) reached 15–30% of the total number of cases. However, with introduction of new anticoagulants in 2001, this percentage decreased to 1–2% [3], and, in recent years, to 0.7–1.7% [4]. Long-term use of anticoagulants is necessary to prevent thromboembolic complications in patients with high risk of thromboembolism. For a long time, vitamin K antagonists (a coumarin derivative warfarin, acenocumarol, and phenindione) and indirect thrombin inhibitors (heparins) have been used as drugs to prevent the occurrence of thromboembolic complications [5,6]. However, despite its effectiveness, coumarin or heparins therapy has some limitations. The heparins must be administered parenterally, and the administration of heparins is carried out under the control of activated partial thromboplastin time (APTT), which should increase by 1.5–2 times from the initial level. A coumarin derivative, warfarin, is characterized by a delayed therapeutic effect (36–72 h after administration, with the development of the maximum effect 5–7 days after the beginning of use) [7]. In addition, there is a need for regular therapeutic drug monitoring with control of the international normalized ratio (INR) indicator at a safe level within two or three times, which entails additional economic burdens on health systems [8]. A significant disadvantage of this group of drugs is the irreversibility of the drug in the event of an overdose [9]. The deviation of the INR from the permissible limits, both in the lower and in higher directions, is prognostically unfavorable. In the first case, the therapeutic effect of anticoagulant therapy will not be achieved. In the second case, the risk of hemorrhagic complications increases [10]. Balancing the effectiveness and safety of anticoagulant therapy is a difficult task in real clinical practice. Genetically determined features of individual’s enzyme systems involved in drug metabolism make significant contributions to their effectiveness and safety [11]. Direct oral anticoagulants (DOACs) such as dabigatran, rivaroxaban, apixaban, and edoxaban have become an alternative to vitamin K antagonists since they do not have the limitations that are inherent in warfarin [12]. DOACs have a predictable pharmacokinetic profile and are free of the disadvantages that are inherent in vitamin K antagonists. However, it is necessary to take into account the pharmacogenetic characteristics of the individual that can affect the effectiveness and safety of the DOACs use.

## 2. Dabigatran

Dabigatran etexilate is the first DOAC that has a direct reversible inhibitory effect on thrombin [13,14]. Thrombin acts as a catalyst for the conversion of factors V, VIII, and XI in blood clotting cascade; it also catalyzes conversion of fibrinogen to fibrin and factor XIII to factor XIIIa, which contributes to the stabilization of fibrin [15]. In addition, thrombin activates GPCR receptors, which leads to conformational changes in platelets and promotes their aggregation. This leads to the release of even more clotting factors and the formation of more thrombin [16].

After entering the human body as an inactive precursor (prodrug), dabigatran etexilate quickly turns into an active metabolite—dabigatran. Dabigatran reversibly binds to the active center of the thrombin molecule, preventing thrombin-mediated activation of the clotting factors. An important feature of dabigatran is that it can inactivate thrombin, even if it is in a bound state with fibrin [17]. The maximum concentration (Cmax) of dabigatran in plasma and, accordingly, anticoagulant action, is observed as early as 0.5–2 h after oral administration [18]. The half-life (T 1/2) of dabigatran after a single dose is 11 h, but with regular intake it increases to 12–14 h, which allows for twicedailydabigatranetexilatedosing [19]. Approximately one third of the dabigatran circulating in blood binds to proteins. The drug is excreted unchanged from the body: 85%—in the urine (via the kidneys); 15%—in the bile (via the liver) [20,21]. 

It is important that dabigatran etexilate is not metabolized by cytochrome P450 isoenzymes of the liver and does not change their activity. The CES1 and CES2 enzymes are human liver carboxylesterases that hydrolyze various xenobiotics and endogenous substrates using ester or amide bonds. The conversion of dabigatran etexylate to dabigatran depends more on the activity of CES1 than on the activity of CES2 [22,23,24].

Glycoprotein P (P-gp) is an ATP-dependent transporter that is involved in the transfer of substrate molecules across membranes of expressing cells and components (regardless of concentration gradient) [25,26]. P-gp is widely present in human body tissues and plays a leading role in the pharmacokinetics of dabigatran etexilate, which is a substrate for P-gp [13]. It is necessary to take into account drug interaction when prescribing dabigatran etexilate with P-gp inhibitors (verapamil, amiodarone, carvedilol, quinidine, spironolactone, nicardipine, propafenone, atorvastatin, clarithromycin, erythromycin, fluoroquinolones, ketoconazole, intraconazole, cyclosporine, fluoxetine, paroxetine, pentazocine, ritonavir, lopinavir, grapefruit juice, and others), as this leads to a decrease in its effectiveness, increased absorption of these drugs, inhibition of their excretion, and increased penetration through barriers. This leads to an increase in the concentration of P-gp substrate drugs in the blood and tissues and increases the risk of adverse drug reactions (ADRs). Bernier M. et al. (2019) revealed development of bleeding in 30.4% of patients taking P-gp inhibitors together with dabigatran [27]. On the contrary, drugs that are inducers of P-gp (rifampicin, morphine, dexamethasone, retinoids, barbiturates, nicotine, diphenin, isoniazid, carbamazepine, caffeine, diazepam, diphenhydramine, tricyclic antidepressants, phenytoin, and ethanol), when used with dabigatran, increase the activity of P-gp, inhibit dabigatran absorption and penetration through barriers, and increase its elimination. This leads to a decrease in the concentration of P-gp substrate drug and reduces its effectiveness. It is also important to take into account that simultaneous use of substrates and P-gp inhibitors increases the risk of developing congenital anomalies in fetuses [28].

In addition to CES1 and ABCB1, which affect the biotransformation of dabigatran etexylate and the effectiveness of active dabigatran, glucuronidation enzymes UGT2B15, UGT1A9, and UGT2B7 also participate in its metabolism (elimination). Their activity reflects safety of using dabigatran [29]. The main and most promising enzyme involved in the elimination of dabigatran is UGT2B15. When prescribing dabigatran etexilate, it is important to consider its interaction with the drugs that are metabolized by UGT2B15. By interacting competitively with the enzyme, these drugs (including, for example, acetaminophen, loratadine, lorazepam, oxazepam, morphine, or valproic acid) can slow down the metabolism of dabigatran [30,31], and its concentration will increase, increasing the risk of ADRs. 

**Figure 1:**
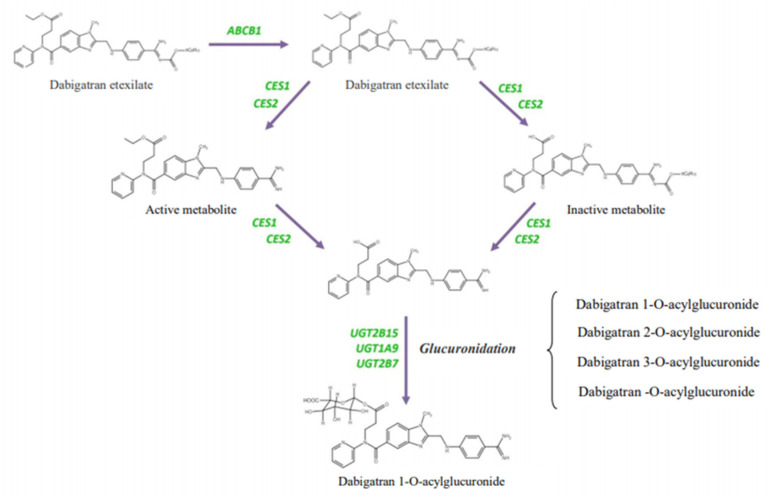
Genes involved in the metabolism of dabigatran etexilate [32].

To date, the *CES1* and *ABCB1* genes have been shown to have an important effect on the metabolism of dabigatran etexilate, and single-nucleotide variants (SNVs) in these two loci probably play a key role (Figure 1) [32]. There have been many studies conducted worldwide to find out whether the search for SNVs in *CES1* and *ABCB1* genes can explain some of inter-individual variability in the concentrations of the active metabolite dabigatran in the blood of humans, and the *UGT2B15* gene may be a potential candidate gene for safety studies of dabigatran. Paré G. et al. (2013) investigated the SNV of the *CES1* gene to assess the inter-individual profile of the efficacy and safety of dabigatran as part of the RE-LY (Randomized Evaluation of LongTerm Anticoagulant Therapy) study [33]. Carriage of minor allele G (rs2244613) of CES1 gene occurred in 32.8% of patients and was associated with minimal concentrations of dabigatran in the blood and, consequently, with a lower risk of bleeding (*p* < 9 × 10^−8^) [33]. Dimatteo C. et al. (2016) found an association of rs8192935 of the *CES1* gene with a lower concentration of dabigatran in blood plasma (*p* = 0.023). Carriers of allele T showed significantly lower concentrations of dabigatran in blood plasma than carriers of the homozygous CC genotype, which reduces the risk of hemorrhagic complications. Overall, the average plasma concentration of dabigatran was higher in patients with the CC genotype (86.3 ng/dL) than in patients with the allele T (62.1 ng/dL). At the same time, there was no significant effect of rs4148738 of the *ABCB1* gene on concentrations of dabigatran in blood [34].

Gouin-Thibault I. et al. (2017) [26] evaluated the effect of clarithromycin on the pharmacokinetics of dabigatran in 60 healthy male volunteers selected for *ABCB1* genotype (20 homozygous carriers of SNVs, 20 heterozygous and 20 homozygous carriers of the wild-type allele for haplotype 2677–3435). The results of the study area under the curve (AUC) were 77% for dabigatran. The *ABCB1* genotype did not significantly affect the pharmacokinetics of dabigatran: the AUC ratio in carriers of studied SNVs and wild-type allele carriers was 1.27 (95% confidence interval (CI) 0.84–1.92), but clarithromycin administration led to a twofold increase in the AUC for dabigatran, regardless of the *ABCB1* genotype: and was 2.0 (95% CI 1.15–3.60) [29]. 

Shi J. et al. (2016) studied the effect of the SNVs of the *CES1* gene and the gender of patients on the effectiveness of dabigatran using several in vitro approaches. Thus, 104 biopsy samples obtained from the liver of patients of various racial backgrounds were examined for carriers of three SNVs: rs2244613, rs8192935, and rs71647871 or G428A, also referred to as G143E, which is a variant of the CES1 enzyme with reduced enzymatic activity. The study showed that G143E is a SNV with reduced metabolism for dabigatran. The activity of CES1 enzyme was significantly higher in female liver samples than in male liver samples. The data obtained by the authors indicate that the studied SNVs of the *CES1* gene and the gender of patients are important risk factors contributing to the variability of the pharmacokinetics of dabigatran etexilate in humans. A personalized approach to treatment with dabigatran etexilate should be based on identifying patient-specific genetic changes in the *CES1*. This approach can potentially improve the effectiveness and safety of pharmacotherapy with this drug [24].

The activity of glucuronidation enzymes depends on the SNVs of their encoding genes. To date, we have not found any works that studythe association of carriers of the *UGT* family genes with the metabolism of dabigatran in humans. However, we can assume that this may change its concentration in blood plasma of patients. This hypothesis is based on previous studies describing associations of the *UGT2B15* gene’s SNVs’ carrier status with the concentration of drugs that are metabolized in a similar way to dabigatran. He X. et al. (2009) found that carriership of allele A (rs1902023) of the *UGT2B15* gene is associated with a decrease in oxazepam clearance. In other words, in patients with this allele, glucuronidation of xenobiotics is slower, and the concentration of drugs in blood plasma increases, thereby increasing the risk of developing ADRs [35]. A similar change in the glucuronidation of drugs in carriers of these SNVs is shown for other drugs that are metabolized in a similar way (lorazepam [31], acetominophen [36], tamoxifen [37], valproic acid [38]). In the study of pharmacokinetics of cypoglitazarus, Stringer F. et al. (2013) showed that patients homozygous for UGT2B15*2 (rs1902023 G > T) had significantly higher concentrations of this drug in the blood compared to patients carrying UGT2B15*1 genotypes/*2 or UGT2B15*1/*1 [39]. Thus, carriership of rs1902023 (UGT2B15*2) of the *UGT2B15* gene is associated with delayed glucuronidation and is an important predictor of interindividual variability in drug clearance. Therefore, this effect can have a significant impact on metabolism of dabigatran as substrate of the UGT2B15 enzyme.

## 3. Rivaroxaban

Rivaroxaban is the first direct factor Xa inhibitor. The pharmacokinetics of rivaroxaban donot share the disadvantages of vitamin K antagonists. However, the pharmacokinetics and pharmacogenetics of rivaroxaban are variable. This can affect both the effectiveness and safety of anticoagulant therapy. Rivaroxaban inhibits platelet activation and fibrin clot formation by direct, selective and reversible inhibition of factor Xa in both intrinsic and extrinsic coagulation pathways. Factor Xa, as part of the prothrombinase complex, also composed of factor Va, calcium ions, factor II, and phospholipids, catalyzes the conversion of prothrombin to thrombin. Thrombin activates platelets and catalyzes the conversion of fibrinogen to fibrin. Thus, factor Xa is a coagulation factor that acts at the point of convergence of internal and external pathways in the blood coagulation system. It catalyzes the breakdown of prothrombin and is, therefore, critical for thrombin generation. It is important to note that rivaroxaban inhibits free prothrombinase- and clot-associated factor Xa without directly affecting platelet aggregation [40]. This distinguishes rivaroxaban from indirect inhibitors of factor Xa, which do not inhibit factor Xa associated with the prothrombinase complex [41].

When taken orally, rivaroxaban reaches its maximum plasma concentration after 2–4 h. The absolute bioavailability of rivaroxaban for dosages of 10 mg is relatively high (80–100%) and does not depend on food intake [42,43]. 

Under fasting conditions, oral bioavailability of rivaroxaban at dosages of 20 mg decreases to 66%. When using drugs at a dosage of 20 mg with food, the AUC increases to 39%. This indicates an almost absolute absorption and, at the same time, a high oral bioavailability of rivaroxaban. The connection with plasma proteins reaches 92–95%. Because of this high plasma protein binding, rivaroxaban is not removed during dialysis [44].

**Figure 2:**
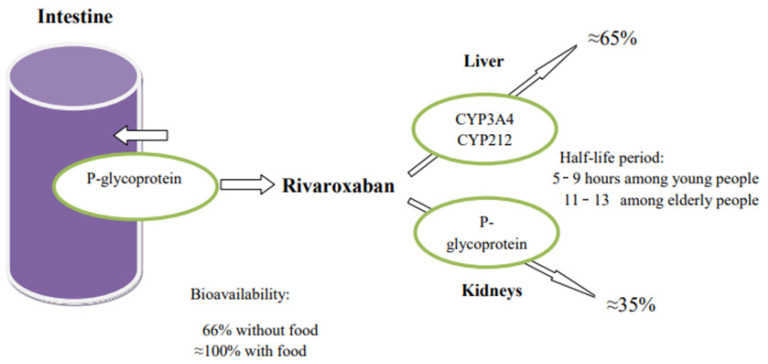
Enzymes involved in the metabolism of rivaroxaban.

Rivaroxaban is eliminated from the body in various ways, of which three are main ones. Approximately 36% of the dose is excreted unchanged by the kidneys through active transporter-mediated secretion by P-gp and BCRP (ABCG2). In addition, 14% of the dose is eliminated by hydrolysis of amide bonds, and 32% of the dose is eliminated via oxidative metabolic pathways. Liver isoenzymes CYP3A4 and CYP3A5 are responsible for the metabolism about 18%—and CYP2J2, about 14%—of the dose (Figure 2) [45,46].

The level of rivaroxaban, when administered concomitantly with midazolam (a CYP3A4 substrate), is reduced by an average of 11% compared with rivaroxaban alone. The following drugs moderately alter the level of rivaroxaban: erythromycin (a moderate inhibitor of CYP3A4/P-gp; an increase of 34%); clarithromycin (potent CYP3A4/mild P-gp inhibitor; 54% increase); fluconazole (moderate CYP3A4, a possible inhibitor of BCRP (ABCG2); an increase of 42%). A significant increase in blood levels and strength of action of rivaroxaban was demonstrated when used simultaneously with drugs that are potent inhibitors of the CYP3A4 enzyme and P-gp/BCRP transporter proteins (ABCG2) as well as potential inhibitors of CYP2J2 enzyme. Specifically, the use of ketoconazole at a dosage of 400 mg once a day leads to an increase in rivaroxaban levels by 158% (95% CI: 136–182%); the use of ritonavir increases the level of rivaroxaban by 153% (95% CI: 134–174%) [47].

The expression of rivaroxaban transporter proteins may be influenced by SNVs of the *ABCB1* gene, but information on their clinical significance is inconsistent. The systematic review and meta-analysis by Xie Q. et al. (2018) showed that Cmax was lower in carriers of *ABCB1* rs1045642 CC than carriers of TT, and carriers of rs2032582 GG than carriers of A/T allele, and AUC0–∞ was lower in rs1045642 CC carriers than in TT carriers [48]. According to Gouin-Thibault I. et al. (2017), *ABCB1* polymorphism cannot be considered as a significant determinant of individual variability in pharmacokinetics of rivaroxaban, and combined use of P-gp/CYP3A4 inhibitor clarithromycin with rivaroxaban may require caution in patients at risk of overdose, as it leads to a two-fold increase in AUC genotype *ABCB1* [26].

Sychev D. et al. (2019) found no significant differences in peak steady-state concentration of rivaroxaban between mutant haplotypes and wild haplotypes of the *ABCB1* gene [49]. Similar results were published by Sennesael A.-L. et al. (2018), who revealed thatSNVs 1236 C> T, −2677 G> T-3435, C> T and 1199 G> A of the *ABCB1* gene did not significantly affect the intracellular accumulation of rivaroxaban compared to wild-type protein. These results suggest that *ABCB1* SNVs examined in the present study are unlikely to contribute to individual variability in plasma rivaroxaban concentrations [50]. At same time, it was found that the use of strong inhibitors and inducers of P-gp should be avoided in patients taking rivaroxaban [26,51].

The study of BCRP protein encoded by the *ABCG2* gene, which, like P-gp, provides absorption and excretion of rivaroxaban from intestinal lumen and renal tubules, seems to be promising. The *ABCG2* gene is increasingly recognized as an important mediator of drug transport in the intestine and renal tubules [52], and its SNVs affect the decrease in BCRP substrate transport in the case of co-administration of rivaroxaban and other drugs [53]. The most studied SNV in this gene, Q141K (rs2231142), is associated with a decrease in BCRP activity and, consequently, with a decrease in the activity of its drug substratetransport [54]. This SNV has not yet been studied in the context of the pharmacogenetics of rivaroxaban; however, in an experimental mouse model, the absence of P-gp (*ABCB1*) and BCRP (*ABCG2*) was associated with significantly reduced drug clearance [55].

Metabolism of rivaroxaban in the liver is carried out by the cytochrome P450 isoenzymes 3A4 (*CYP3A4*) and 2J2 (*CYP2J2*), as well as by mechanisms independent of CYP [47]. To date, more than 50 SNVs of the *CYP3A4* gene have been discovered, associated with different levels of activity of the 3A4 isoenzyme. Associations between *CYP3A4* SNVs’ carriership and changes in pharmacological response have been described for atorvastatin, simvastatin, sacrolimus, and fentanyl [56]. Information on the change in pharmacological response of rivaroxaban in the literature available to us was not found. At the same time, it was found that the use of strong inhibitors and inducers of CYP3A4 enzyme and P-gp should be avoided in patients taking rivaroxaban [51]. For example, “old” antiepileptic drugs (AEDs) that act on cytochrome P450 enzymes, and especially on CYP3A4, such as phenobarbital, phenytoin, and carbamazepine, are more likely to significantly reduce the anticoagulant effect of DOACs (especially rivaroxaban, apixaban, and edoxaban). New AEDs that do not significantly affect CYP or P-gp, such as lamotrigine or pregabalin, are unlikely to affect the effectiveness of DOACs. Zonisamide and lacosamide, which do not significantly interfere with in vitro CYP activity, may have a safe profile. However, their effect on P-gp has not yet been studied. Levetiracetam only has a potential effect on P-gp activity, so it may also be safe [57].

The study of the effect of a potent P-gp inhibitor, cyclosporin, and its combination with a moderate CYP3A inhibitor, fluconazole, on the pharmacokinetics of rivaroxaban and CYP3A activity (compared with baseline) showed that cyclosporine increased the average exposure of rivaroxaban by 47%. The combination of rivaroxaban with fluconazole increased the average exposure of rivaroxaban by 86% and the maximum concentration of fluconazole by 115%. This effect was significantly stronger than that observed in the control group that received rivaroxaban with fluconazole alone [58].

The high clinical significance of the interaction of rivaroxaban with other drugs is shown in a systematic review and meta-analysis of studies in which patients with atrial fibrillation were randomized to groups taking DOACs or warfarin, stratified by the number of concomitant drugs [59]. Polypharmacy was significantly associated with poor outcomes and reduced the benefit in terms of risk of major bleeding in patients receiving rivaroxaban, especially in the presence of drugs that modulate P-gp/CYP3A4.

Additionally, about ten different SNVs for the *CYP2J2* gene are known, but their clinical role was mainly studied in the context of coronary heart disease (CAD) and arterial hypertension, since isoenzyme CYP2J2 encoded by this gene plays a role in the metabolism of arachidonic acid [60].

## 4. Apixaban

Apixaban is a direct oral reversible and highly selective factor Xa inhibitor that does not require antithrombin III for antithrombotic activity [61,62]. Apixaban inhibits both free and clot-associated factor Xa and prothrombinase activity, which inhibits clot growth [63]. By inhibiting factor Xa, apixaban reduces the formation of thrombin and the development of blood clots. It has no direct effect on platelet aggregation, but indirectly inhibits thrombin-induced platelet aggregation [64].

Absorption of apixaban occurs mainly in the small intestine and gradually decreases as it passes through it [65]. For oral doses of up to 10 mg, the absolute bioavailability of apixaban is about 50% due to incomplete absorption [66,67] and the first passage through the liver [68,69]. Apixaban Cmax in plasma is reached 3–4 h after oral administration [70,71]. Binding of apixaban to blood plasma proteins, mainly albumin, is about 87% [72]. After oral administration, unchanged apixaban is the main drug component in human blood plasma without presence of active circulating metabolites [67]. Excretion of apixaban involves several pathways, including metabolism in the liver, as well as excretion by unchanged metabolites in the bile and kidneys, and direct intestinal excretion [73].Metabolic pathways of apixaban include O-demethylation, hydroxylation, and sulfation of hydroxylated O-demethylapixaban [67]. At same time, metabolism mainly occurs through isoenzymes CYP3A4/5 of liver cytochrome P450, with an insignificant participation of isoenzymes CYP1A2, CYP2C8, CYP2C9, CYP2C19 and CYP2J2 [68]. 

The role of non-functional allele G (rs776746) of the *CYP3A5* gene is the most studied. At the same time, in heterozygous carriers (genotype AG), the metabolism of apixaban is moderately reduced due to carriership of one non-functional allele G, and, in heterozygous carriers (CYP3A5*3, genotype GG), the CYP3A5 isoenzyme is not expressed. This constitutes a risk factor for ADRsdevelopment (in particular, bleeding) when taking apixaban [74]. Ueshima S. et al. (2017) found that patients with AF and a homozygous TT genotype (rs77674) of the *CYP3A5* gene may have decreased blood apixaban concentrations compared to patients with CC and CT genotypes. Therefore, carriership of allele T may be associated with an increased clearance of apixaban [74]. However, this study was conducted on Asian patients, which does not allow extrapolation of the results to other racial and ethnic groups. 

The highest risk of developing apixaban-induced ADRscaused by a slowdown in the metabolism of the drug in the liver, especially when combined with drug-inhibitors of CYP3A5 isoenzyme, in homozygous carriers of non-functional alleles CYP3A5*2 (rs28365083), CYP3A5*3 (rs776746), CYP3A5*6 (rs10264272), CYP3A5*7 (rs41303343), CYP3A5*8 (rs55817950), CYP3A5*9 (rs28383479), CYP3A5*10 or CYP3A5*3K (rs41279854), CYP3A5*11 (rs72552791), CYP3A5*3D (rs56244447), CYP3A5*3F (rs28365085), *CYP3A5*3705C>T (H30Y) (rs28383468), *CYP3A5*7298C>A(S100Y) (rs41279857). Among them, the most common is non-functional allele CYP3A5*3 (rs776746). In terms of phenotypes, individuals are “expressors” of CYP3A5 if they carry at least one CYP3A5*1 allele, and “non-expressors” if not. It should be noted that the frequency of *CYP3A5* gene SNVs’ carriership varies significantly depending on patients’ ethnicity. For example, most Europeans are not expressors, while many people of African descent are CYP3A5 expressors [64,75]. Higher concentrations of the active component of drugs, metabolized with participation of isoenzyme CYP3A5, in blood plasma are higher in non-expressors of CYP3A5 compared with expressors [76]. Apixaban dosing should be conducted withcaution, and requires monitoring of ADRs in non-CYP3A5-expressing patients (homozygous carriers of the above non-functional alleles). Co-administration of apixaban with other drugs metabolized with the participation of the CYP3A5 isoenzyme should be avoided in non-expressors.

The study of SNVs of the *CYP3A5* gene was conducted among 200 postmenopausal women who had an episode of venous thromboembolism and more than 500 comparable control groups. It is known that oral estrogen intake increases the risk of venous thromboembolism in all women (odds ratio (OR)—4.5; CI: 2.6–7). Compared with women who did not receive oral estrogens, the OR for venous thromboembolism in users of oral estrogens was 3.8 (CI: 2.1–6.7) among women who did not have the common (wild) CYP3A5*1 allele encoding a highly functional isoenzyme CYP3A5, and 30.0 (CI: 4.4–202.9) among patients with this allele (interaction test *p* = 0.04) [77]. This is important to consider when prescribing apixaban to postmenopausal women.

Carriership of low-functional alleles CYP1A2*1C (rs2069514), CYP1A2*1K −729C>T (rs12720461), CYP1A2*1K −739T>G (rs2069526), CYP1A2*3 (rs56276452), CYP1A2*4A (rs56276455) and CYP1A*4A (rs28399424) of the *CYP1A2* gene leads to a decrease in activity of the CYP1A2 isoenzyme. This may be of clinical significance in long-term therapy with apixaban in homozygous carriers of low- or non-functional alleles of the *CYP3A5* gene, due to the cumulative risk and disruption of the auxiliary pathway of apixaban metabolism in the liver with the participation of the isoenzyme CYP1A2. This reduces the metabolism of the drug and increases the risk of ADRs. In addition, in carriers of CYP1A2*1C (rs2069514), concomitant use of apixaban with inhibitors of the isoenzyme CYP1A2 may slow down the breakdown of caffeine, which can lead to overstimulation by caffeine. Alternatively, carriership of the highly functional allele CYP1A2*1F (rs762551) can lead to an acceleration of apixaban metabolism. Smoking is a well-known CYP1A2 activator (especially in CYP1A2*1F carriers). It leads to a more rapid degradation of drugs metabolized with the participation of the CYP1A2 isoenzyme, and the possibility of insufficient concentration of drugs in the body to obtain significant therapeutic benefits [78]. 

Carriers of SNVs of the *CYP2C9* gene can metabolize drugs in different ways. From a clinical point of view, carriership of the following SNVs is of interest: rs1057910 (two variants that encode the “wild-type”CYP2C9*1 allele and the non-functional CYP2C9*3 allele), as well as rs1799853, rs9332131, rs72558190, and rs72558 (non-functional variants CYP2C9*2, CYP2C9*6, CYP2C9*15, and CYP2C9*25, respectively). In particular, the carriership of non-functional alleles CYP2C9*2 and CYP2C9*3 should be taken into account in the case of co-administration of apixaban and clopidogrel, which inhibits the CYP2C9 isoenzyme in sufficiently high doses. This may affect the metabolism of drugs that are metabolized with the participation of the isoenzyme CYP2C9, and patients who are homozygous carriers of non-functional alleles of the *CYP2C9* gene (poor metabolizers—PMs) are likely to be at greater risk of ADRs (in particular, the risk of bleeding) when taking clopidogrel and apixaban [51].

Some of the major metabolic pathways of apixaban include o-demethylation, hydroxylation, and sulfation, with o-demethylapixaban sulfate being the main metabolite [66]. A potentially important pharmacogenomic metabolic pathway is via sulfotransferases (SULT) SULT1A1 and SULT1A2, which are responsible for sulfation of o-demethyl-apixaban to o-demethyl-apixaban sulfate [79,80]. The SULT1A1 enzyme is more efficient than SULT1A2 in the sulfation of o-demethyl-apixaban [81]. O-demethyl-apixaban is the most well-known metabolite; it accounts for 25% of the estimated active apixaban [67]. It is important to know that o-demethyl-apixaban sulfate does not have any inhibitory activity against factor Xa, which may contribute to the anticoagulant efficacy of apixaban [81]. Three important SNVs of the *SULT1A1* gene have been described: SULT1A1*1 (wild type), SULT1A1*2 (rs9282861), and SULT1A1*3 (rs1801030) [80]. The Vmax of all three allelic variants of the *SULT1A1* gene (SULT1A1*1 >SULT1A1*3 >SULT1A1*2) varies, and this explains the differences in sulfation of active apixaban. The SULT1A1*3 variant has a moderate potential to influence the anticoagulant effect of apixaban, whereas SULT1A1*2 has low potential to influence apixaban metabolism [82]. These different alloenzymes have different enzymatic efficiencies and can lead to different concentrations of metabolites and variations in the anticoagulant efficacy of apixaban [83]. However, the effect of common genetic variants of the *SULT1A1* gene on apixaban metabolism in patients has not yet been formally studied [51].

## 5. Edoxaban

Edoxaban is a selective, direct and reversible inhibitor of activated blood coagulation factor X (F Xa), a serine protease responsible for thrombin formation. Edoxaban is used to prevent stroke in nonvalvular AF, and to treat deep vein thrombosis and PE [84,85,86]. It binds to both free FXa and free FXa in the prothrombinase complex, thus causing a dose-dependent decrease in thrombin formation [87].

Edoxaban is characterized by a linear, predictable pharmacokinetics profile [88]. After oral administration, edoxaban reaches peak plasma concentrations (C max) within 1–2 h [89]. The half-life (T1/2) of edoxaban is approximately 10–14 h [88]. Edoxaban is absorbed mainly in the upper gastrointestinal tract; approximately 13% is absorbed in the large intestine [90]. In an in vitro study, five phase 1 metabolites of edoxaban were found in the human liver microsomes: M-1, M-4, M-5, M-6 and a hydroxylated metabolite at the N-dimethylcarbamoyl group of edoxaban (hydroxymethylenedoxaban) (M-7) [91]. Formation of a metabolite M-4, unique for humans, is catalyzed by CES1, which is present in the human liver microsomes and in the cytosol. Cytochrome P450 (CYP) 3A4 isoenzyme mediates the formation of M-5 and hydroxymethylenedoxaban in the presence of nicotinamide adenine dinucleotide phosphate (NADPH). It is assumed that M-8, minor metabolite, arises spontaneously (non-enzymatically) via an intermediary, hydroxymethylenedoxaban, which is formed via CYP3A4/5 [92].

The second phase of edoxaban metabolism is mediated by glucuronidation to form N-glucuronide metabolite (M-3). This metabolite has not been quantified. Three metabolites (M-4, M-6 and M-8) have anticoagulant activity with half-maximum inhibitory concentration (IC50) values for anti-FXa 1.8 nM (M-4), 6.9 nM (M-6) and 2.7 nM (M-8). The IC 50 value of edoxaban for anti-FXa is 3 nM [93]. However, due to its low content and high protein binding (80%), it is expected that most abundant metabolite M-4 will not make a significant contribution to the overall pharmacological activity of edoxaban in patients with at least a moderate decline in renal function [94].

**Figure 3:**
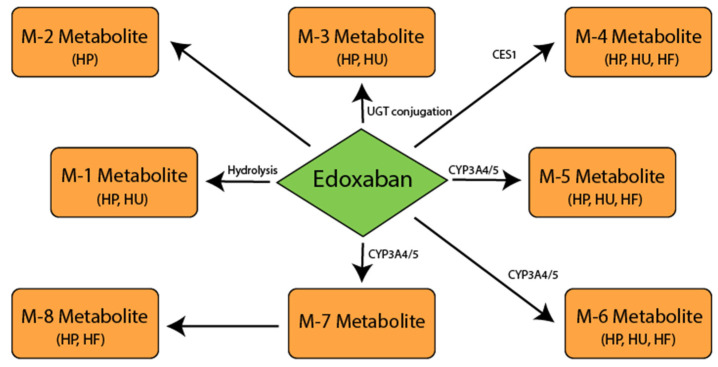
Postulated metabolic pathway for edoxaban. Carboxylesterase 1 (CES1); cytochrome P450 isoenzyme 3A4/5 (CYP3A4/5); human feces (HF); human plasma (HP); human urine (HU); metabolite (M); 5′-diphospho-glucuronosyltransferase (UGT).

The second phase of edoxaban metabolism is mediated by glucuronidation to form N-glucuronide metabolite (M-3). This metabolite has not been quantified. Three metabolites (M-4, M-6 and M-8) have anticoagulant activity with half-maximum inhibitory concentration (IC50) values for anti-FXa 1.8 nM (M-4), 6.9 nM (M-6) and 2.7 nM (M-8) (Figure 3). The IC 50 value of edoxaban for anti-FXa is 3 nM [92]. However, due to its low content and high protein binding (80%), it is expected that most abundant metabolite M-4 will not make a significant contribution to the overall pharmacological activity of edoxaban in patients with at least a moderate decline in renal function [94]. Other metabolites are present in even smaller amounts and (in the absence of liver cytochrome P450 inducers) do not significantly contribute to the total anticoagulant activity of drugs. None of the metabolic pathways alone contribute more than 10% to the total clearance of edoxoban [92].Edoxaban is a substrate for P-gp and is not a substrate for other transporters such as anion transport polypeptide (OATPs), 1B1, or organic cation transporters (OATs) 2 [95].

**Figure 4:**
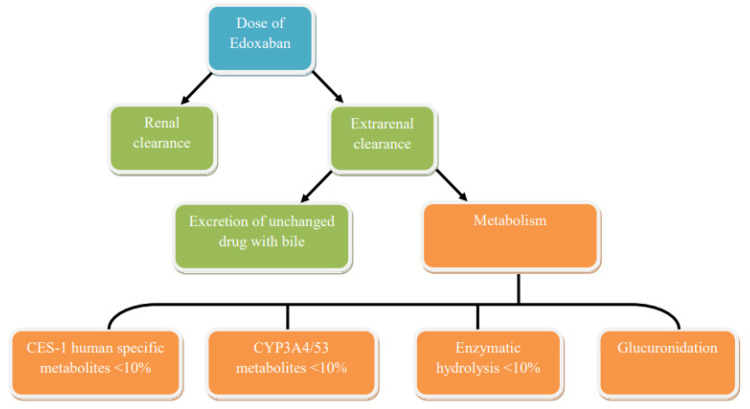
Chart of pathways for the elimination of edoxaban in adults.

Edoxaban is mainly excreted unchanged in urine and through the secretion of the biliary tract with feces [92]. Renal clearance of unchanged drugs is approximately 50% of total clearance, and the remaining 50% of non-renal clearance occurs due to metabolism and secretion of the biliary tract (Figure 4). As previously described, edoxaban is metabolized by the enzymes CES1 (<10%), CYP3A4 (<10%) and by glucuronidation, but metabolism is a minor route of elimination of edoxaban in patients with normal renal function. 

Therefore, inhibitors or inducers of these enzymes are unlikely to have clinically significant interactions with edoxaban. However, drug interaction studies have been performed to investigate the effect of CYP3A4 inhibitors on the pharmacokinetics of edoxaban. In addition, the effects of other drugs that could be administered concurrently with edoxaban were evaluated. Since edoxaban is a substrate of the P-gp efflux transporter, several studies have been carried out on the interactions of drugs with inhibitors, substrates and inducers of P-gp. The effect of co-administration of P-gp inhibitors was an increase in the effect of edoxaban (maximum observed drug concentration in plasma C max and area under the curve (AUC) of concentration versus time), but the increase was less than two times. P-gp inhibitors and potent CYP3A4/5 inhibitors (e.g., ketoconazole, erythromycin) do not result in greater increases in exposure compared to mild P-gp inhibitors (e.g., verapamil) or mild inhibitors (e.g., cyclosporine) CYP3A4/5. This confirms that the metabolism of CYP3A4/5 is not the main route of elimination of edoxaban [96,97].

Co-administration of ketoconazole (P-gp inhibitor; potent CYP3A4 inhibitor) increased the single dose peak and overall exposure to edoxaban by 89% and 87%, respectively [98]. However, co-administration of oral quinidine (P-gp and OCT2 transporter inhibitor; potent CYP2D6 inhibitor) increased the single dose peak and 24-h exposure of oral edoxaban by 85% and 77%, respectively [99].

Co-administration of sustained-release verapamil (P-gp inhibitor (main effect); moderate CYP3A4 inhibitor) increased the peak and 24-h exposure of single doses of edoxaban by 53% [99].

Co-administration of erythromycin (P-gp inhibitor; moderate CYP3A4 inhibitor) increased the peak and total exposure of single doses of edoxaban by 68% and 85%, respectively [91]. Co-administration of cyclosporine (P-gp inhibitor, OATP1B1 and BCRP; weak inhibitor of CYP3A4) increased both the peak and total exposure of single doses of edoxaban by 74% and 73%, respectively [98]. Co-administration of dronedarone (a P-gp inhibitor) increased the peak and total exposure of single doses of edoxaban by 46% and 85%, respectively [99].

The administration of amiodarone (P-gp inhibitor; moderate CYP2C9 inhibitor, weak CYP2D6 inhibitor) to patients receiving edoxaban for 3 days of once daily administration increased the peak and total exposure of single doses of edoxaban by 66% and 40%, respectively [99]. This is important to remember because amiodarone has a long half-life, reaching an average of 58 days [100]. Rifampicin, an inducer of P-gp (strong CYP3A4 inducer; moderate inducer of CYP2B6, 2C8, 2C9, 2C19; inhibitor of P-gp, OATP1B1, OATP1B3), reduced the total exposure to edoxaban by about 34% after 7 days of dosing, without affecting its peak exposure [101]. Co-administration of digoxin (P-gp substrate) increased the C max of edoxaban by 16% without significantly affecting overall exposure or renal clearance at the steady state [99].

At the same time, atorvastatin (OATP1B1 and OATP1B3 substrate; weak CYP3A4 inhibitor), when taken together with edoxaban, does not affect the peak or total exposure of edoxaban [99]. Co-administration of naproxen and edoxaban also had no effect on the peak and total exposure of edoxaban. However, it led to an increase in the duration of bleeding compared with each drug administered separately. Co-administration of naproxen increased the baseline-adjusted bleeding time ratio by 72% on day 2 compared with edoxaban alone (90% CI: 139.3–213.3). In contrast, concomitant administration of edoxaban with naproxen increased the equivalent bleeding time by 22% compared with naproxen alone (90% CI: 98.1–151.0) [102]. Naproxen reduced the baseline-adjusted platelet aggregation coefficient on the second day of co-administration by 69.89% (90% CI: 68.20–71.62), while edoxaban itself did not affect platelet aggregation.

Co-administration of high doses of aspirin (325 mg) increased the stationary peak and total exposure of edoxaban by 34% and 30%, respectively, and decreased renal clearance by 17%, possibly due to inhibition of active renal secretion. Co-administration of low-dose aspirin (100 mg) did not affect the peak or total exposure of edoxaban either after a single dose or with stable use (90% CI: 80–125%). Co-administration of edoxaban and aspirin at low (100 mg) or high (325 mg) doses resulted in an additive effect in terms of the increased bleeding time. The anticoagulant effects of edoxaban were not affected by the simultaneous administration of aspirin. Co-administration of low doses of aspirin (100 mg) did not significantly affect the INR, prothrombin time (PTI), activated partial thromboplastin time (APTT), or intrinsic FXa activity [102]. Enoxaparin did not affect the peak and total exposure of edoxaban with simultaneous dosing or with an interval of 12 h. Co-administration of edoxaban at a dose of 60 mg and subcutaneous enoxaparin at a dose of 1 mg/kg led to an increase in the effect on the parameters of the analysis of thrombin formation compared to any of the drugs introduced separately. The effect was generally not additive, with the exception of the delay in thrombin formation and the time to peak. The effect on anti-FXa with the simultaneous use of both drugs was additive [103].

Candidate genes influencing the concentration of edoxaban are genes encoding key enzymes of its metabolism: *CES1*, *CYP3A4/5*, *ABCB1* [55] and, to a lesser extent, *SLCO1B1* [104].Edoxaban and its active metabolite M4 are substrates for P-gp encoded by the *ABCB1* (*MDR1*) gene and the organic anion carrier protein OATP1B1 encoded by the *SLCO1B1* gene. The pharmacogenomics analysis combined genotype and concentration-time data in 458 healthy volunteers in 14 completed phase 1 studies. The SNVs effect of the *ABCB1* gene (rs1045642: C3435T) and *SLCO1B1* gene (rs4149056: T521C) on pharmacokinetics parameters of edoxaban was studied. Although some pharmacological inhibitors of P-gp and OATP1B1 increased exposure to edoxaban, neither C3435T (rs1045642) of the *ABCB1* gene, nor T521C (rs4149056) of the *SLCO1B1* gene, affected the pharmacokinetics of edoxaban. However, a slight increase in M4 exposure was observed in carriers of minor allele C* of the *SLCO1B1* gene [104].

Only a limited amount of edoxaban is metabolized by liver cytochrome P450 isoenzymes (less than 4%) [105]. Metabolites M4 and M1 are formed during the hydrolysis of edoxaban with the participation of the CES1 enzyme encoded by the *CES1* gene, while M6 is formed through metabolism with the participation of the CYP3A4/5 isofermet, encoded by the *CYP3A5* gene [92]. Analysis of genomic associations showed that SNVs of the *CES1* gene affect the plasma levels of dabigatran [106]. So far, no studies have been found on the effect of carriership of the studied SNVs of the *CES1* gene on the pharmacokinetics of edoxaban. However, this may be promising in terms of personalized selection of DOACs.

There is probably a high risk of developing edoxaban-induced adverse reactions due to a slowdown in the metabolism of the drug in the liver when combined with drug inhibitors of the CYP3A5 isoenzyme in homozygous carriers of non-functional alleles of the *CYP3A5* gene. Thus, in the non-expressing CYP3A5 patients (homozygous carriers of the above non-functional alleles), dosing of edoxaban should be calculated with caution and requires monitoring of the risk of bleeding. Co-administration of edoxaban with other drugs metabolized with the participation of the isoenzyme CYP3A5 should be avoided in non-expressors, including antipsychotics (olanzapine), antiestrogens (tamoxifen), antineoplastics (irinotecan, docetaxel and vincristine), immunomodulatory agents (tacrolimus), antiplatelet agents (clopidogrel), antihypertensive agents (nifedipine, amlodipine, felodipine and verapamil), antiviraldrugs (indinavir, nelfinavir, ritonavir and saquinavir), HMG-CoA reductase inhibitors (atorvastatin), antibiotics (clarithromycin), steroids (testosterone, estradiol, progesterone and androstenedione), antimalarial drugs (mefloquine, artemether and lumefantrine) [107].

## 6. Discussion

Unfortunately, the larger multicenter randomized trials using pharmacogenetics (PGx) data of DOACs were not found. Perhaps this is due to the fact that PGx of DOACs has been studied only in recent years. We suggest that such trials are conducted for different races and ethnic groups. The PGx variants can affect the pharmacokinetics of the DOACs, especially co-administration DOACs with other drugs (Table 1 and Table 2), which are prescribed to patients with cerebrovascular diseases and comorbid disorders.

The pharmacodynamics of DOACs are not discussed in the review, because we analyzed only its PGx and pharmacokinetics. However, we agree that discussion about the PGx and pharmacodynamics of the drugs is very important for future reviews.

We undoubtedly anticipate the value of creating a DOACs dose calculator using pharmacogenomics data. It is, however, necessary to carry out additional large, randomized trials using PGx data to clarify the role of candidate genes SNVs, which cause a predisposition to altering the pharmacokinetics of DOACs in humans. This is very important, because PGx testing can be used for therapeutic selection.

## 7. Conclusions

The results of the fundamental and clinical studies of DOACs conducted to date indicate an undeniable influence of genome changes on the pharmacokinetics of DOACs. However, the studies need to be continued. There is a need to plan and conduct larger studies across various ethnic groups with the inclusion of sufficient associative genetic studies of the number of patients in each of the treatment groups with well-defined phenotypes. Additional work is needed to transfer research results into real clinical practice using the results of PGx testing and taking into account genomic variations for the selection of DOACs, and their starting and target dosages, which is especially important if long-term pharmacotherapy is required.

## Figures and Tables

**Table 1:** Drug–drug interaction of the DOACs.

Direct Oral Anticoagulants (DOACs)	Drugs That Increase DOACs’ Concentration in the Serum	Drugs That Decrease DOACs’ Concentration in the Serum
Apixaban	Olanzapine Tamoxifen Irinotecan Docetaxel Vincristine Meflohin Artemether Lumefantrine Tacrolimus Cyclosporine Chlorphenamine Terfenadine Astemizole Clopidgrel Nifedipine Amlodipine Felodipine Verapamil Indinavir Nelfinavir Ritonavir Saquinavir Atorvastatin Cerivastatin Clarothromycin Testosterone Estradiol Progesterone	Pifampicin
Dabigatran etexilate	Ketoconazole Chinidine	Rifampicin
Rivaroxaban	n/a	Rifampicin Phenytoine Carbamazepine Phenobarbital
Edoxaban	Amiodarone Ketoconazole Chinidine Erythromycin Cyclosporine	n/a

**Table 2:** The candidate genes predisposing to changes in the DOACs’ metabolism.

Drugs	Enzymes Involved in the Metabolism of DOACs	Candidate Genes Involved in the Metabolism of DOACs
Apixaban	Isoenzyme 3A4/3A5 of the hepatic cytochrome 450 Sulfotransferase 1	*CYP3A4/CYP2A5* *SULT1A1*
Dabigatran etexilate	Isoenzymes 1A2, 2C8, 2C9, 2C19 and 2J2 of the hepatic cytochrome 450	*CYP1A2, CYP2C8, CYP2C9, CYPC19, CYP2J2*
Rivaroxaban	Sulfotransferase 1	*CES1*
Edoxaban	Isoenzymes 3A4/3A5 and 2J2 of the hepatic cytochrome 450	*CYP3A4/CYP3A5, CYP2J2*

## References

[1] Heit J.A. (2005). Venous thromboembolism: Disease burden, outcomes and risk factors. J. Thromb. Haemost..

[2] Feigin V.L., Lawes C.M., A Bennett D., Barker-Collo S.L., Parag V. (2009). Worldwide stroke incidence and early case fatality reported in 56 population-based studies: A systematic review. Lancet Neurol..

[3] Falck-Ytter Y., Francis C.W., Johanson N.A., Curley C., Dahl O.E., Schulman S., Ortel T.L., Pauker S.G., Colwell C.W. (2012). Prevention of VTE in Orthopedic Surgery Patients: Antithrombotic Therapy and Prevention of Thrombosis, 9th ed: American College of Chest Physicians Evidence-Based Clinical Practice Guidelines. Chest.

[4] Pedersen A.B., Mehnert F., Sorensen H.T., Emmeluth C., Overgaard S., Johnsen S.P. (2014). The risk of venous thromboembolism, myocardial infarction, stroke, major bleeding and death in patients undergoing total hip and knee replacement. Bone Jt. J..

[5] Ezekowitz M.D., Bridgers S.L., James K.E., Carliner N.H., Colling C.L., Gornick C.C., Krause-Steinrauf H., Kurtzke J.F., Nazarian S.M., Radford M.J. (1992). Warfarin in the Prevention of Stroke Associated with Nonrheumatic Atrial Fibrillation. N. Engl. J. Med..

[6] Simonneau G., Sors H., Charbonnier B., Page Y., Laaban J.-P., Azarian R., Laurent M., Hirsch J.-L., Ferrari E., Bosson J.-L. (1997). A Comparison of Low-Molecular-Weight Heparin with Unfractionated Heparin for Acute Pulmonary Embolism. The THESEE Study Group. Tinzaparine ou Heparine Standard: Evaluations dans l’Embolie Pulmonaire. N. Engl. J. Med..

[7] Holford N.H.G. (1986). Clinical Pharmacokinetics and Pharmacodynamics of Warfarin. Understanding the dose-effect relationship. Clin. Pharmacokinet..

[8] Shendre A., Dillon C., Beasley T.M., Parmar G.M., A Limdi N. (2018). Influence of Age on Warfarin Dose, Anticoagulation Control, and Risk of Hemorrhage. Pharmacother. J. Hum. Pharmacol. Drug Ther..

[9] Shatzel J.J., Daughety M.M., Prasad V., Deloughery T.G. (2017). Reversal of warfarin era thinking. J. Intern. Med..

[10] Barnes G.D. (2019). Predicting the Quality of Warfarin Therapy: Reframing the Question. Thromb. Haemost..

[11] Wu A.H. (2018). Pharmacogenomic-guided dosing for warfarin: Too little too late?. Pers. Med..

[12] Schulman S., Kearon C., Kakkar A.K., Mismetti P., Schellong S., Eriksson H., Baanstra D., Schnee J., Goldhaber S.Z. (2009). Dabigatran versus Warfarin in the Treatment of Acute Venous Thromboembolism. N. Engl. J. Med..

[13] Stangier J., Rathgen K., Stähle H., Gansser D., Roth W. (2007). The pharmacokinetics, pharmacodynamics and tolerability of dabigatran etexilate, a new oral direct thrombin inhibitor, in healthy male subjects. Br. J. Clin. Pharmacol..

[14] Hankey G.J., Eikelboom J.W. (2011). Dabigatran Etexilate: A new oral thrombin inhibitor. Circulation.

[15] Goldsack N.R., Chambers R.C., Dabbagh K., Laurent G.J. (1998). Molecules in focus Thrombin. Int. J. Biochem. Cell Biol..

[16] Davie E.W., Kulman J.D. (2006). An Overview of the Structure and Function of Thrombin. Semin. Thromb. Hemost..

[17] Comin J., Kallmes D. (2012). Dabigatran (Pradaxa). Am. J. Neuroradiol..

[18] Gelosa P., Castiglioni L., Tenconi M., Baldessin L., Racagni G., Corsini A., Bellosta S. (2018). Pharmacokinetic drug interactions of the non-vitamin K antagonist oral anticoagulants (NOACs). Pharmacol. Res..

[19] Comuth W.J., Henriksen L.Ø., van de Kerkhof D., Husted S.E., Kristensen S.D., de Maat M.P., Münster A.-M.B. (2018). Comprehensive characteristics of the anticoagulant activity of dabigatran in relation to its plasma concentration. Thromb. Res..

[20] Antonijevic N.M., Zivkovic I.D., Jovanovic L.M., Matic D.M., Kocica M.J., Mrdovic I.B., Kanjuh V.I., Culafic M.D. (2017). Dabigatran—Metabolism, Pharmacologic Properties and Drug Interactions. Curr. Drug Metab..

[21] Fawzy A.M., Lip G.Y.H. (2019). Pharmacokinetics and pharmacodynamics of oral anticoagulants used in atrial fibrillation. Expert Opin. Drug Metab. Toxicol..

[22] Satoh T., Hosokawa M. (2006). Structure, function and regulation of carboxylesterases. Chem. Interact..

[23] Ghosh S., Natarajan R. (2001). Cloning of the Human Cholesteryl Ester Hydrolase Promoter: Identification of Functional Peroxisomal Proliferator-Activated Receptor Responsive Elements. Biochem. Biophys. Res. Commun..

[24] Shi J., Wang X., Nguyen J.-H., Bleske B.E., Liang Y., Liu L., Zhu H.-J. (2016). Dabigatran etexilate activation is affected by the CES1 genetic polymorphism G143E (rs71647871) and gender. Biochem. Pharmacol..

[25] Chen Z., Shi T., Zhang L., Zhu P., Deng M., Huang C., Hu T., Jiang L., Li J. (2016). Mammalian drug efflux transporters of the ATP binding cassette (ABC) family in multidrug resistance: A review of the past decade. Cancer Lett..

[26] Gouin-Thibault I., Delavenne X., Blanchard A., Siguret V., Salem J.E., Narjoz C., Gaussem P., Beaune P., Funck-Brentano C., Azizi M. (2017). Interindividual variability in dabigatran and rivaroxaban exposure: Contribution of ABCB 1 genetic polymorphisms and interaction with clarithromycin. J. Thromb. Haemost..

[27] Bernier M., Lancrerot S.L., Rocher F., Van-Obberghen E.K., Olivier P., Lavrut T., Parassol-Girard N., Drici M.D. (2019). Major bleed-ing events in octagenarians associated with drug interactions between dabigatran and P-gp inhibitors. J. Geriatr. Cardiol..

[28] Daud A.N.A., Bergman J.E.H., Bakker M.K., Wang H., Kerstjens-Frederikse W.S., De Walle H.E.K., Groen H., Bos J.H.J., Hak E., Wilffert B. (2015). P-Glycoprotein-Mediated Drug Interactions in Pregnancy and Changes in the Risk of Congenital Anomalies: A Case-Reference Study. Drug Saf..

[29] Ebner T., Wagner K., Wienen W. (2010). Dabigatran Acylglucuronide, the Major Human Metabolite of Dabigatran: In Vitro Formation, Stability, and Pharmacological Activity. Drug Metab. Dispos..

[30] UniProt UDP-glucuronosyltransferase 2B15. UniProt Knowledgebase. www.uniprot.org/uniprot/P54855.

[31] Chung J., Cho J., Yu K.-S., Kim J., Jung H.-R., Lim K., Jang I.-J., Shin S.-G. (2005). Effect of the genotype on the pharmacokinetics, pharmacodynamics, and drug interactions of intravenous lorazepam in healthy volunteers. Clin. Pharmacol. Ther..

[32] Savinova A.V., Shnayder N.A., Petrova M.M., Nasyrova R.F. (2020). Promising areas of research on the pharmacogenetics of dabigatran etexilate. Pharmacokinet. Pharm..

[33] Paré G., Eriksson N., Lehr T., Connolly S., Eikelboom J., Ezekowitz M.D., Axelsson T., Haertter S., Oldgren J., Reilly P. (2013). Genetic Determinants of Dabigatran Plasma Levels and Their Relation to Bleeding. Circulation.

[34] Dimatteo C., D’Andrea G., Vecchione G., Paoletti O., Cappucci F., Tiscia G.L., Buono M., Grandone E., Testa S., Margaglione M. (2016). Pharmacogenetics of dabigatran etexilate interindividual variability. Thromb. Res..

[35] He X., Hesse L.M., Hazarika S., Masse G., Harmatz J.S., Greenblatt D.J., Court M.H. (2009). Evidence for oxazepam as aninvivoprobe of UGT2B15: Oxazepam clearance is reduced byUGT2B15D85Y polymorphism but unaffected byUGT2B17deletion. Br. J. Clin. Pharmacol..

[36] Court M.H., Zhu Z., Masse G., Duan S.X., James L.P., Harmatz J.S., Greenblatt D.J. (2017). Race, Gender, and Genetic Polymorphism Contribute to Variability in Acetaminophen Pharmacokinetics, Metabolism, and Protein-Adduct Concentrations in Healthy African-American and European-American Volunteers. J. Pharmacol. Exp. Ther..

[37] Savelyeva M.I., Urvantseva I.A., Ignatova A.K., Panchenko J.S., Poddubnaya I.V. (2017). Pharmacogenetic features of the phase II bio-transformation of tamoxifen: A systematic review. Pharmacogenomics.

[38] Ethell B.T., Anderson G.D., Burchell B. (2003). The effect of valproic acid on drug and steroid glucuronidation by expressed human UDP-glucuronosyltransferases. Biochem. Pharmacol..

[39] Stringer F., Ploeger B.A., De Jongh J., Scott G., Urquhart R., Karim A., Danhof M. (2013). Evaluation of the Impact of UGT Polymorphism on the Pharmacokinetics and Pharmacodynamics of the Novel PPAR Agonist Sipoglitazar. J. Clin. Pharmacol..

[40] Perzborn E., Roehrig S., Straub A., Kubitza D., Mueck W., Laux V. (2010). Rivaroxaban: A New Oral Factor Xa Inhibitor. Arter. Thromb. Vasc. Biol..

[41] Turpie A.G. (2007). Oral, Direct Factor Xa Inhibitors in Development for the Prevention and Treatment of Thromboembolic Diseases. Arter. Thromb. Vasc. Biol..

[42] Kubitza D., Becka M., Voith B., Zuehlsdorf M., Wensing G. (2005). Safety, pharmacodynamics, and pharmacokinetics of single doses of BAY 59-7939, an oral, direct factor Xa inhibitor. Clin. Pharmacol. Ther..

[43] Kubitza D., Becka M., Wensing G., Voith B., Zuehlsdorf M. (2005). Safety, pharmacodynamics, and pharmacokinetics of BAY 59-7939—An oral, direct Factor Xa inhibitor—After multiple dosing in healthy male subjects. Eur. J. Clin. Pharmacol..

[44] Weinz C., Buetehorn U., Daehler H.-P., Kohlsdorfer C., Pleiss U., Sandmann S., Schlemmer K.-H., Schwarz T., Steinke W. (2005). Pharmacokinetics of BAY 59-7939—An oral, direct Factor Xa inhibitor—In rats and dogs. Xenobiotica.

[45] Weinz C., Schwarz T., Kubitza D., Mueck W., Lang D. (2009). Metabolism and Excretion of Rivaroxaban, an Oral, Direct Factor Xa Inhibitor, in Rats, Dogs, and Humans. Drug Metab. Dispos..

[46] CHMP Assessment Report For Xarelto. https://www.ema.europa.eu/en/documents/assessment-report/xarelto-epar-public-assessment-report_en.pdf.

[47] Mueck W., Kubitza D., Becka M. (2013). Co-administration of rivaroxaban with drugs that share its elimination pathways: Pharmacokinetic effects in healthy subjects. Br. J. Clin. Pharmacol..

[48] Xie Q., Xiang Q., Mu G., Ma L., Chen S., Zhou S., Hu K., Zhang Z., Cui Y., Jiang J. (2018). Effect of ABCB1 Genotypes on the Pharmacokinetics and Clinical Outcomes of New Oral Anticoagulants: A Systematic Review and Meta-analysis. Curr. Pharm. Des..

[49] Sychev D., Minnigulov R., Bochkov P., Ryzhikova K., Yudina I., Lychagin A., Morozova T. (2019). Effect of CYP3A4, CYP3A5, ABCB1 Gene Polymorphisms on Rivaroxaban Pharmacokinetics in Patients Undergoing Total Hip and Knee Replacement Surgery. High Blood Press. Cardiovasc. Prev..

[50] Sennesael A.-L., Panin N., Vancraeynest C., Pochet L., Spinewine A., Haufroid V., Elens L. (2018). Effect of ABCB1 genetic polymorphisms on the transport of rivaroxaban in HEK293 recombinant cell lines. Sci. Rep..

[51] Kanuri S.H., Kreutz R.P. (2019). Pharmacogenomics of Novel Direct Oral Anticoagulants: Newly Identified Genes and Genetic Variants. J. Pers. Med..

[52] Cusatis G., Sparreboom A. (2008). Pharmacogenomic importance of ABCG2. Pharmacogenomics.

[53] Cusatis G., Gregorc V., Li J., Spreafico A., Ingersoll R.G., Verweij J., Ludovini V., Villa E., Hidalgo M., Sparreboom A. (2006). Pharmacogenetics of ABCG2 and Adverse Reactions to Gefitinib. J. Natl. Cancer Inst..

[54] Woodward O.M., Tukaye D.N., Cui J., Greenwell P., Constantoulakis L.M., Parker B.S., Rao A., Köttgen M., Maloney P.C., Guggino W.B. (2013). Gout-causing Q141K mutation in ABCG2 leads to instability of the nucleotide-binding domain and can be corrected with small molecules. Proc. Natl. Acad. Sci. USA.

[55] O’Connor C.T., Kiernan T.J., Yan B.P. (2017). The genetic basis of antiplatelet and anticoagulant therapy: A pharmacogenetic review of newer antiplatelets (clopidogrel, prasugrel and ticagrelor) and anticoagulants (dabigatran, rivaroxaban, apixaban and edoxaban). Expert Opin. Drug Metab. Toxicol..

[56] Solus J.F., Arietta B.J., Harris J.R., Sexton D.P., Steward J.Q., McMUNN C., Ihrie P., Mehall J.M., Edwards T.L., Dawson E.P. (2004). Genetic variation in eleven phase I drug metabolism genes in an ethnically diverse population. Pharmacogenomics.

[57] Galgani A., Palleria C., Iannone L.F., De Sarro G., Giorgi F.S., Maschio M., Russo E. (2018). Pharmacokinetic Interactions of Clinical Interest Between Direct Oral Anticoagulants and Antiepileptic Drugs. Front. Neurol..

[58] Brings A., Lehmann M., Foerster K.I., Burhenne J., Weiss J., Haefeli W.E., Czock D. (2019). Perpetrator effects of ciclosporin (P-glycoprotein inhibitor) and its combination with fluconazole (CYP3A inhibitor) on the pharmacokinetics of rivaroxaban in healthy volunteers. Br. J. Clin. Pharmacol..

[59] Harskamp R.E., Teichert M., Lucassen W.A.M., Van Weert H.C.P.M., Lopes R.D. (2019). Impact of Polypharmacy and P-Glycoprotein- and CYP3A4-Modulating Drugs on Safety and Efficacy of Oral Anticoagulation Therapy in Patients with Atrial Fibrillation. Cardiovasc. Drugs Ther..

[60] Wu S.-N., Zhang Y., Gardner C.O., Chen Q., Li Y., Wang G.-L., Gao P.-J., Zhu D.-L. (2007). Evidence for Association of Polymorphisms in CYP2J2 and Susceptibility to Essential Hypertension. Ann. Hum. Genet..

[61] Luettgen J.M., Knabb R.M., He K., Pinto D.J.P., Rendina A.R. (2010). Apixaban inhibition of factor Xa: Microscopic rate constants and inhibition mechanism in purified protein systems and in human plasma. J. Enzym. Inhib. Med. Chem..

[62] Ansell J. (2007). Factor Xa or thrombin: Is factor Xa a better target?. J. Thromb. Haemost..

[63] Jiang X., Crain E.J., Luettgen J.M., Schumacher W.A., Wong P.C. (2009). Apixaban, an oral direct factor Xa inhibitor, inhibits human clot-bound factor Xa activity in vitro. Thromb. Haemost..

[64] Savinova A.V., Petrova M.M., Shnayder N.A., Bochanova E.N., Nasyrova R.F. (2020). Pharmacokinetics and Pharmacogenetics of Apixaban. Ration. Pharmacother. Cardiol..

[65] Byon W., Nepal S., Schuster A.E., Shenker A., Frost C.E. (2018). Regional Gastrointestinal Absorption of Apixaban in Healthy Subjects. J. Clin. Pharmacol..

[66] Vakkalagadda B., Frost C., Byon W., Boyd R.A., Wang J., Zhang D., Yu Z., Dias C., Shenker A., LaCreta F. (2016). Effect of Rifampin on the Pharmacokinetics of Apixaban, an Oral Direct Inhibitor of Factor Xa. Am. J. Cardiovasc. Drugs.

[67] Raghavan N., Frost C.E., Yu Z., He K., Zhang H., Humphreys W.G., Pinto D., Chen S., Bonacorsi S., Wong P.C. (2008). Apixaban Metabolism and Pharmacokinetics after Oral Administration to Humans. Drug Metab. Dispos..

[68] Wang L., Zhang D., Raghavan N., Yao M., Nirmala R., Frost C.A., Maxwell B.D., Chen S.-Y., He K., Goosen T.C. (2009). In Vitro Assessment of Metabolic Drug-Drug Interaction Potential of Apixaban through Cytochrome P450 Phenotyping, Inhibition, and Induction Studies. Drug Metab. Dispos..

[69] Zhang D., He K., Herbst J.J., Kolb J., Shou W., Wang L., Balimane P.V., Han Y.-H., Gan J., Frost C.E. (2013). Characterization of Efflux Transporters Involved in Distribution and Disposition of Apixaban. Drug Metab. Dispos..

[70] Frost C., Wang J., Nepal S., Schuster A., Barrett Y.C., Mosqueda-Garcia R., Reeves R.A., LaCreta F. (2013). Apixaban, an oral, direct factor X a inhibitor: Single dose safety, pharmacokinetics, pharmacodynamics and food effect in healthy subjects. Br. J. Clin. Pharmacol..

[71] Frost C., Nepal S., Wang J., Schuster A., Byon W., Boyd R.A., Yu Z., Shenker A., Barrett Y.C., Mosqueda-Garcia R. (2013). Safety, pharmacokinetics and pharmacodynamics of multiple oral doses of apixaban, a factor X a inhibitor, in healthy subjects. Br. J. Clin. Pharmacol..

[72] He K., Luettgen J.M., Zhang D., He B., Grace J.E., Xin B., Pinto D.J.P., Wong P.C., Knabb R.M., Lam P.Y.S. (2011). Preclinical pharmacokinetics and pharmacodynamics of apixaban, a potent and selective factor Xa inhibitor. Eur. J. Drug Metab. Pharmacokinet..

[73] Wang X., Mondal S., Wang J., Tirucherai G., Zhang D., Boyd R.A., Frost C. (2014). Effect of Activated Charcoal on Apixaban Pharmacokinetics in Healthy Subjects. Am. J. Cardiovasc. Drugs.

[74] Ueshima S., Hira D., Fujii R., Kimura Y., Tomitsuka C., Yamane T., Tabuchi Y., Ozawa T., Itoh H., Horie M. (2017). Impact of ABCB1, ABCG2, and CYP3A5 polymorphisms on plasma trough concentrations of apixaban in Japanese patients with atrial fibrillation. Pharm. Genom..

[75] SNPedia CYP3A5. https://www.snpedia.com/index.php/CYP3A5.

[76] Kang R.-H., Jung S.-M., Kim K.-A., Lee D.-K., Cho H.-K., Jung B.-J., Kim Y.-K., Kim S.-H., Han C., Lee M.-S. (2009). Effects of CYP2D6 and CYP3A5 Genotypes on the Plasma Concentrations of Risperidone and 9-Hydroxyrisperidone in Korean Schizophrenic Patients. J. Clin. Psychopharmacol..

[77] Canonico M., Bouaziz E., Carcaillon L., Verstuyft C., Guiochon-Mantel A., Becquemont L., Scarabin P.-Y. (2008). Synergism between Oral Estrogen Therapy and Cytochrome P450 3A5*1 Allele on the Risk of Venous Thromboembolism among Postmenopausal Women. J. Clin. Endocrinol. Metab..

[78] SNPedia CYP1A2. https://www.snpedia.com/index.php/CYP1A2.

[79] Sweezy T., A Mousa S. (2014). Genotype-guided use of oral antithrombotic therapy: A pharmacoeconomic perspective. Pers. Med..

[80] Carlini E.J., Raftogianis R.B., Wood T.C., Jin F., Zheng W., Rebbeck T.R., Weinshilboum R.M. (2001). Sulfation pharmacogenetics:SULT1A1 and SULT1A2 allele frequencies in Caucasian, Chinese and African-American subjects. Pharmacogenetics.

[81] Wang L., Raghavan N., He K., Luettgen J.M., Humphreys W.G., Knabb R.M., Pinto D.J., Zhang D. (2009). Sulfation of O-Demethyl Apixaban: Enzyme Identification and Species Comparison. Drug Metab. Dispos..

[82] Nagar S., Walther S., Blanchard R.L. (2006). Sulfotransferase (SULT) 1A1 Polymorphic Variants *1, *2, and *3 Are Associated with Altered Enzymatic Activity, Cellular Phenotype, and Protein Degradation. Mol. Pharmacol..

[83] Raftogianis R.B., Wood T.C., Otterness D.M., Van Loon J.A., Weinshilboum R.M. (1997). Phenol Sulfotransferase Pharmacogenetics in Humans: Association of CommonSULT1A1Alleles with TS PST Phenotype. Biochem. Biophys. Res. Commun..

[84] Fuji T., Fujita S., Kawai Y., Nakamura M., Kimura T., Fukuzawa M., Abe K., Tachibana S. (2015). Efficacy and safety of edoxaban versus enoxaparin for the prevention of venous thromboembolism following total hip arthroplasty: STARS J-V. Thromb. J..

[85] Fuji T., Wang C.-J., Fujita S., Kawai Y., Kimura T., Tachibana S. (2014). Safety and Efficacy of Edoxaban, an Oral Factor Xa Inhibitor, for Thromboprophylaxis After Total Hip Arthroplasty in Japan and Taiwan. J. Arthroplast..

[86] Fuji T., Fujita S., Kawai Y., Nakamura M., Kimura T., Kiuchi Y., Abe K., Tachibana S. (2014). Safety and ef-ficacy of edoxaban in pa-tients undergoing hip fracture surgery. Thromb Res..

[87] Samama M.M., Mendell J., Guinet C., Le Flem L., Kunitada S. (2012). In vitro study of the anticoagulant effects of edoxaban and its effect on thrombin generation in comparison to fondaparinux. Thromb. Res..

[88] Ogata K., Mendell-Harary J., Tachibana M., Masumoto H., Oguma T., Kojima M., Kunitada S. (2010). Clinical Safety, Tolerability, Pharmacokinetics, and Pharmacodynamics of the Novel Factor Xa Inhibitor Edoxaban in Healthy Volunteers. J. Clin. Pharmacol..

[89] Matsushima N., Lee F., Sato T., Weiss D., Mendell J. (2013). Bioavailability and Safety of the Factor Xa Inhibitor Edoxaban and the Effects of Quinidine in Healthy Subjects. Clin. Pharmacol. Drug Dev..

[90] Parasrampuria D.A., Kanamaru T., Connor A., Wilding I., Ogata K., Shimoto Y., Kunitada S. (2015). Evaluation of regional gastrointestinal absorption of edoxaban using the enterion capsule. J. Clin. Pharmacol..

[91] Parasrampuria D.A., Truitt K.E. (2016). Pharmacokinetics and Pharmacodynamics of Edoxaban, a Non-Vitamin K Antagonist Oral Anticoagulant that Inhibits Clotting Factor Xa. Clin. Pharmacokinet..

[92] Bathala M.S., Masumoto H., Oguma T., He L., Lowrie C., Mendell J. (2012). Pharmacokinetics, Biotransformation, and Mass Balance of Edoxaban, a Selective, Direct Factor Xa Inhibitor, in Humans. Drug Metab. Dispos..

[93] Daiichi Sankyo, Inc. Savaysa (Edoxabantosylate): FDA Cardiovascular and Renal Drugs Adviso-ry Committee Briefing Document. NDA 206316. http://www.fda.gov/AdvisoryCommittees/CommitteesMeetingMaterials/Drugs/CardiovascularandRenalDrugsAdvisoryCommittee/ucm420703.htm.

[94] Jönsson S., Simonsson U.S.H., Miller R., Karlsson M.O. (2015). Population pharmacokinetics of edoxaban and its main metabolite in a dedicated renal impairment study. J. Clin. Pharmacol..

[95] Mikkaichi T., Yoshigae Y., Masumoto H., Imaoka T., Rozehnal V., Fischer T., Okudaira N., Izumi T. (2014). Edoxaban Transport via P-Glycoprotein Is a Key Factor for the Drug’s Disposition. Drug Metab. Dispos..

[96] FDA Center for Drug Evaluation Research FDA Draft Guidance for Industry: Drug Interaction Studies—Study Design, Data Analysis, Implications for Dosing, and Labeling Recommendations. http://www.fda.gov/downloads/Drugs/Guidances/ucm292362.pdf.

[97] Flockhart D.A. Drug Interactions: Cytochrome P450 Drug Interaction Table. Indiana University School of Medicine. https://static.medicine.iupui.edu/divisions/clinpharm/content/p450_Table_Oct_11_2009.pdf.

[98] Parasrampuria D.A., Mendell J., Shi M., Matsushima N., Zahir H., Truitt K. (2016). Edoxaban drug–drug interactions with ketoconazole, erythromycin, and cyclosporine. Br. J. Clin. Pharmacol..

[99] Mendell J., Zahir H., Matsushima N., Noveck R., Lee F., Chen S., Zhang G., Shi M. (2013). Drug-Drug Interaction Studies of Cardiovascular Drugs Involving P-Glycoprotein, an Efflux Transporter, on the Pharmacokinetics of Edoxaban, an Oral Factor Xa Inhibitor. Am. J. Cardiovasc. Drugs.

[100] Cordarone® (Amiodarone HCl) Tablets: Full Prescribing Information. Wyeth Pharmaceuticals Inc., a Subsidiary of Pfizer Inc.; Philadelphia. http://labeling.pfizer.com/showlabeling.aspx?id=93.

[101] Mendell J., Chen S., He L., Desai M., Parasramupria D.A. (2015). The Effect of Rifampin on the Pharmacokinetics of Edoxaban in Healthy Adults. Clin. Drug Investig..

[102] Mendell J., Lee F., Chen S., Worland V., Shi M., Samama M.M. (2013). The Effects of the Antiplatelet Agents, Aspirin and Naproxen, on Pharmacokinetics and Pharmacodynamics of the Anticoagulant Edoxaban, a Direct Factor Xa Inhibitor. J. Cardiovasc. Pharmacol..

[103] Matsushima N., Halim A.-B., He L., Zhang G., Lee F., Worland V., Mendell J., Zahir H. (2012). Edoxaban administration following enoxaparin: A pharmacodynamic, pharmacokinetic, and tolerability assessment in human subjects. Thromb. Haemost..

[104] Vandell A.G., Lee J., Shi M., Rubets I., Brown K.S., Walker J.R. (2016). An integrated pharmacokinetic/pharmacogenomic analysis of ABCB1 and SLCO1B1 polymorphisms on edoxaban exposure. Pharm. J..

[105] Bounameaux H., Camm A.J. (2014). Edoxaban: An Update on the New Oral Direct Factor Xa Inhibitor. Drugs.

[106] Stangier J. (2008). Clinical Pharmacokinetics and Pharmacodynamics of the Oral Direct Thrombin??Inhibitor Dabigatran Etexilate. Clin. Pharmacokinet..

[107] Umamaheswaran G., Kumar D.K., Adithan C. (2014). Distribution of genetic polymorphisms of genes en-coding drug metabolizing enzymes & drug transporters—A review with Indian perspective. Indian J. Med. Res..

